# Modeling the Generation of Phase-Amplitude Coupling in Cortical Circuits: From Detailed Networks to Neural Mass Models

**DOI:** 10.1155/2015/915606

**Published:** 2015-10-11

**Authors:** Roberto C. Sotero

**Affiliations:** Department of Radiology and Hotchkiss Brain Institute, University of Calgary, Calgary, AB, Canada T3A 2E1

## Abstract

Phase-amplitude coupling (PAC), the phenomenon where the amplitude of a high frequency oscillation is modulated by the phase of a lower frequency oscillation, is attracting an increasing interest in the neuroscience community due to its potential relevance for understanding healthy and pathological information processing in the brain. PAC is a diverse phenomenon, having been experimentally detected in at least ten combinations of rhythms: delta-theta, delta-alpha, delta-beta, delta-gamma, theta-alpha, theta-beta, theta-gamma, alpha-beta, alpha-gamma, and beta-gamma. However, a complete understanding of the biophysical mechanisms generating this diversity is lacking. Here we review computational models of PAC generation that range from detailed models of neuronal networks, where each cell is described by Hodgkin-Huxley-type equations, to neural mass models (NMMs) where only the average activities of neuronal populations are considered. We argue that NMMs are an appropriate mathematical framework (due to the small number of parameters and variables involved and the richness of the dynamics they can generate) to study the PAC phenomenon.

## 1. Introduction

From the theory of signal processing we know that if an input-state-output system is linear its output will have the same frequency content as its inputs. Conversely, in nonlinear systems, the energy at one frequency in the inputs appears at different frequencies in the outputs. This induces cross-frequency coupling (CFC) between any two sources, when the output of one serves as the input to the other [1]. It has been shown that pyramidal cells produce a varied set of intrinsic dynamics based only on the type and compartmental localization of intrinsic conductances [2]. A combination of sodium, potassium, and calcium conductances produces coexistent gamma (~40 Hz) and theta (~6 Hz) rhythms on tonic depolarization. In contrast, combinations of persistent sodium and potassium channels in the soma produce a use-dependent transition between regular spiking at ~10 Hz and a repetitive, brief burst generation at ~20 Hz [2]. Since cortical columns and brain areas generating different brain rhythms are interconnected, the presence of CFC should not be surprising, even if the exact mechanisms responsible for its generation remain imprecise. The question is then whether CFC is only a mechanistic result of the way the brain is constructed or if it also has a role in brain computations. At least six types of CFC have been documented [3, 4]: amplitude-amplitude coupling (AAC), phase-phase coupling (PPC), frequency-frequency coupling (FFC), phase-amplitude coupling (PAC), phase-frequency coupling (PFC), and frequency-amplitude coupling (FAC). PAC, the type of CFC that occurs when the phase of a low frequency oscillation modulates the amplitude of a higher frequency oscillation, has received a lot of attention in the last decade due to its potential relevance for understanding healthy and pathological brain function [5–11]. PAC has been hypothesized to be the carrier mechanism for the interaction of local and global processes and therefore being directly linked to the integration of distributed information in the brain [12]. For instance, it has been suggested that theta-gamma PAC is used as a coding scheme for multi-item short-term memory in the hippocampus, where different spatial information is represented in different gamma subcycles of a theta cycle [13, 14]. Recent experimental evidence also suggests that PAC links local blood-oxygen-level-dependent (BOLD) signals to BOLD correlation across distributed networks [15].

In parallel to the experimental study of the PAC phenomenon, computational models have been proposed in order to clarify the neurobiological mechanism underlying its generation [16–23]. Here we review these models, going from the detailed description of each cell (via the Hodgkin-Huxley formalism) in neuronal networks to neural mass models (NMMs), which are a type of mean field description that focuses on the dynamics of the average activity in a neuronal population while neglecting the second-order statistics (variance and covariances) and from models only focusing on generation of the theta-gamma PAC in the hippocampus to the most recent models capable of simultaneously generating several PAC combinations.

This review is structured as follows. First, in Section 2, we show that there is evidence for at least ten different PAC combinations (of a low and a higher frequency oscillation). Computational models of the PAC phenomenon can be divided into two types: detailed and NMMs. The main characteristics of these two types are briefly discussed in Section 3, followed by two sections describing specific models of both types.

## 2. Experimental Evidence of the Diversity of the PAC Phenomenon

The classic example of PAC was demonstrated in the CA1 region of the hippocampus [24] where the phase of the theta rhythm was found to modulate the power of gamma oscillations. Later studies found that PAC is neither restricted to theta-gamma coupling nor to the hippocampus. For instance, PAC has also been reported in the frontal, posterior, and parietal human cortices during auditory, visual, linguistic, and memory tasks [25–27], in monkey auditory and visual cortices [15, 28, 29] and rodent olfactory bulb [30]. In addition to Bragin et al.'s study [24], other studies have confirmed the existence of theta-gamma coupling in the hippocampus [31–34] and other brain areas [35–44]. Other PAC combinations of low and high frequency rhythms have also been detected: delta-theta [37, 45], delta-alpha [46, 47], delta-beta [44, 46], delta-gamma [34, 35, 38, 41, 44], theta-alpha [46], theta-beta [44, 46], alpha-beta [45], alpha-gamma [15, 26, 27, 35, 46, 48, 49], and beta-gamma [7, 15].

It should be noted that the studies mentioned above do not always use the same frequency values for the boundaries of the different brain rhythms [50] and that sometimes the gamma band is divided into different subbands such as low-gamma, middle-gamma, and fast-gamma, with boundaries that can differ between different studies. Thus, subdivisions of classical bands can potentially increase the number of PAC combinations to be studied. Additionally, a high number of mathematical methods for detecting PAC have been proposed [3, 12, 51–57], each with advantages and caveats, and no gold standard has emerged. Furthermore, those methods are not exempted of spurious results, that is, identifying PAC that is not related to true modulations between neuronal subsystems. These issues (reviewed recently in [58]) are out of the scope of this review, but we mention them here to highlight the fact that the experimental study of the PAC phenomenon is far from being complete and new methods and results in the upcoming years will be necessary to complement, inform, and refine past and future computational models of the phenomenon.

## 3. Detailed Mathematical Models versus Neural Mass Models

There are two main approaches to modeling the dynamics of neuronal populations. One approach is to realistically model each cell in the network, using multiple compartments for the soma, axon, and dendrites. The most prominent example of this approach is the Blue Brain Project [59], which aims to achieve in the next decade a full simulation of human brain dynamics (a network of approximately 86 billion neurons) in a supercomputer. A practical disadvantage of such realistic modeling is that it requires high computational power. For this reason, simplified versions of such models in which only one compartment is taken into account have been used [16, 60]. However, even in this case, the use of such detailed models makes it difficult to determine the influence of each model parameter on the generated average network characteristics. The second approach is based on the use of NMMs, which constitute a class of biophysical models that captures the average activity of neuronal ensembles, rather than modeling each neuron in the network individually [61, 62]. NMMs are described by nonlinear differential equations and can be rigorously obtained from mean field approaches [63–65] after neglecting the second-order moments. For instance, the Wilson-Cowan neural mass model [61] can be obtained from a mean field approximation of two coupled networks of FitzHugh-Nagumo neurons [63]. An alternative way of constructing the NMM formalism is to consider that each neuronal population performs two mathematical operations [62]. The first is the conversion of postsynaptic potentials (PSP) into an average density of action potentials (AP) which is modeled using a sigmoid function. The second operation is the conversion of AP into PSP, which is done by means of a linear convolution with an impulse response function. The Wilson-Cowan model is obtained when the impulse response function is *g*(*t*) = *Ge* ^−*kt*^, which produces a system of first-order differential equations describing the activity in each population. A more recent neural mass model, the Jansen-Rit model [62], is obtained when the impulse response function has the form *g*(*t*) = *Gkte* ^−*kt*^. This results in a system of second-order differential equations describing the dynamics of PSPs in each population. Computational models based on Wilson-Cowan and Jansen-Rit models have provided the mathematical framework for simulating the generation of electrical activity in the brain during resting state [62, 66–71], stimulation [62, 72–74], and disease [67, 75–77].

## 4. Detailed Mathematical Models

Detailed mathematical models of PAC generation [16, 18] have focused on the theta-gamma interaction observed in the hippocampus [24]. These models consist of either purely inhibitory networks [16] or networks with both excitatory and inhibitory cells [18–20] and are based on models previously developed to study the generation of theta and gamma rhythms separately [23].

### 4.1. Inhibitory-Inhibitory (*I*-*I*) Network

A simulated inhibitory network in the hippocampus containing fast and slow interneurons was shown to generate theta-gamma coupling under restricted conditions [16]. The network comprised single compartment neurons modeled with the Hodgkin-Huxley formalism:

(1) $$\begin{array}{c} C\frac{dV_{i}}{dt}=I_{i}-I_{Na}-I_{K}-I_{L}-I_{S}+\eta , \end{array}$$

where index *i* = 1,…, *N*, counts the cells in the network, *I*_*i*_ is the applied current, and *η* is a normally distributed noise. The sodium (*I*_Na_), potassium (*I*_K_), leak (*I*_*L*_), and synaptic (*I*_*S*_) currents are given by

(2) INa=gNam∞3hVi−VNa,IK=gKn4Vi−VK,IL=gLVi−VL,IS=∑j=1Ngs,jNsjVi−Vs.

The cell population (*N* = 100) was divided into half on the basis of fast and slow synaptic dynamics. Synaptic conductances *g*_*s*,*j*_ had one of four possible values depending on the types of the cells connected: fast cell to fast cell, fast cell to slow cell, slow cell to slow cell, and slow cell to fast cell. Connectivity was all to all. Equations for the gating variables *h*, *n*, and *s*, as well as parameter values can be found in [16]. The numerical simulations performed in [16] showed that the model can generate PAC under restricted conditions that included strong connections within the same populations, weaker connections between populations (especially from fast to slow populations), and carefully tuned inputs.

### 4.2. Excitatory-Inhibitory (*E*-*I*) Networks

Hippocampal networks producing theta-gamma PAC also have pyramidal cells. To consider this situation, a model comprising three neuronal populations was proposed in [18] and was shown to produce theta-gamma PAC [23]. The three populations are pyramidal cells, fast-spiking basket cells, and oriens lacunosum-moleculare (O-LM) interneurons. The outputs of the O-LM cells are projected as slow inhibitory postsynaptic potentials (IPSP) onto the distal apical dendrites of pyramidal cells [18].

Basket cells were modeled with a single compartment, using the fast-spiking interneuron model proposed in [78], similar to (1) and (2). O-LM cells were also modeled with a single compartment. In addition to sodium, potassium, leak, and synaptic current, two other currents were considered: the h-current and the A current [17, 18]. Pyramidal cells were modeled by 5 compartments: 1 for basal dendrites, 1 for soma, and 3 for apical dendrites. The equation for each compartment *k* (1,…, 5) is

(3) CEkdVEkdt=Iapp,Ek−INa,Ek−IK,Ek−IL,Ek−Ih,Ek−IA,Ek−Isyn,Ek+Iconn,Ek,

where *I*_conn,*E*_*k*__ is the current due to electrical coupling between compartments. The expressions for the ionic and synaptic currents as well as the parameter values to simulate the model can be found in the supplementary information section in [18]. Different simulations were performed in [18], but the one with the highest number of cells comprised a total of 180 cells. Their results showed that O-LM cells alone can coordinate cell assemblies and that the same theta rhythm can coordinate different cell assemblies with different frequencies in the gamma range [18, 23].

## 5. Neural Mass Models

In this section we review three NMMs that are able to generate PAC. The first two studies [21, 22] are based on the works of Wilson-Cowan and Jansen-Rit and only focus on the generation of one PAC combination. The last study [79] is also based on the Jansen-Rit model but is able to simultaneously generate different PAC combinations.

### 5.1. PAC Generation Using the Wilson-Cowan Model

**Figure 1:**
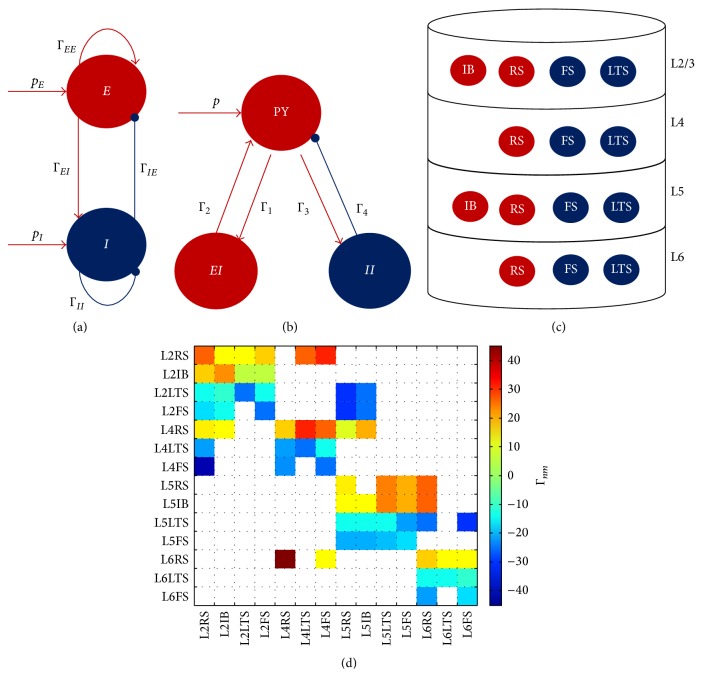
Neural mass models. (a) Wilson-Cowan model (Section 5.1) of two coupled populations, one excitatory (*E*) and one inhibitory (*I*). External inputs to these populations are *p*_*E*_ and *p*_*I*_, and the connectivity parameters are Γ_*EI*_, Γ_*IE*_, Γ_*EE*_, and Γ_*II*_. (b) Jansen-Rit model (Section 5.2) of a cortical column. Three populations are modeled: pyramidal cells (PY), excitatory interneurons (*EI*), and inhibitory interneurons. The connectivity parameters are Γ_1_, Γ_2_, Γ_3_, and Γ_4_, and the input to the model is *p*. (c) Neural mass model of the cortical column comprising 14 populations (Section 5.3) distributed across 4 layers. The excitatory populations are the intrinsically bursting (IB) and the regulatory spiking (RS). The inhibitory population are the low-threshold spiking (LTS) and fast-spiking (FS). The connections between the populations are depicted in (d). Any of the 14 populations can be subjected to an external input. In the three models ((a), (b), and (c)), excitatory populations and connections are depicted in red and inhibitory ones in blue. (d) Connectivity matrix values used for coupling the 14 populations are modeled in (c). Negative values correspond to inhibitory connections and positive values correspond to excitatory ones.

Onslow et al. [21] used the Wilson-Cowan model to study the generation of theta-gamma PAC in a brain region not necessarily restricted to the hippocampus. The model comprises two coupled populations (Figure 1(a)), one excitatory and one inhibitory. The system of first-order differential equations describing the model is

(4) dEtdt=1τEE+1τESpE+ΓEEE−ΓIEI,dItdt=−1τII+1τISpI+ΓEIE−ΓIII,

where *E*(*t*) and *I*(*t*) are the average activity levels of excitatory and inhibitory populations, respectively [61] and *p*_*E*_ and *p*_*I*_ are the external inputs to the two populations. The weight of the connection from the excitatory population to the inhibitory population is Γ_*EI*_ and from the inhibitory to the excitatory population is Γ_*IE*_, and the self-connections are Γ_*EE*_ and Γ_*II*_. *τ*_*E*_ and *τ*_*I*_ are the time constants for each population. The nonlinearity in the model is introduced by means of a sigmoid function:

(5) $$\begin{array}{c} S\left(\;x\;\right)=\frac{S_{0}}{1+e^{r\left(x-v_{0}\right)}}, \end{array}$$

where parameter *r* determines the steepness of the sigmoid curve, *v*_0_ determines the position of the sigmoid function, and *S*_0_ determines the amplitude of the response.

System (4) is capable of producing oscillations due to the reciprocal connections between the two populations. Numerical simulations showed [21] that this model generates gamma oscillations that are locked to a certain phase of theta oscillations when considering oscillatory inputs.

**Figure 2:**
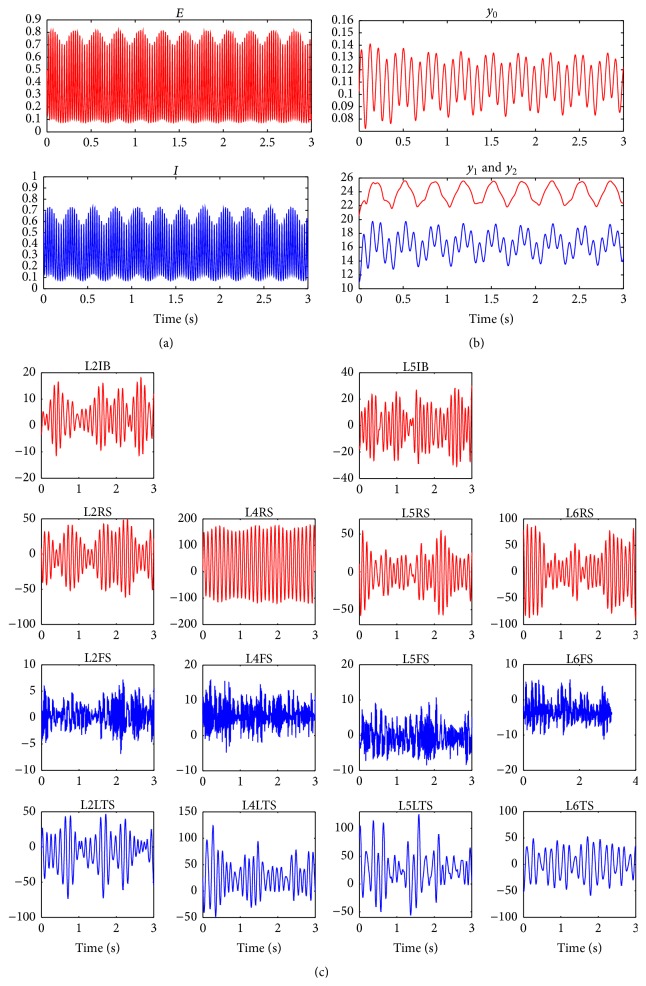
Simulated temporal evolution of the variables of three different neural mass models. (a) Wilson-Cowan model. The phase of a theta oscillation (4 Hz) modulates the amplitude of a gamma oscillation (55 Hz). (b) Jansen-Rit model. The phase of a delta oscillation (3 Hz) modulates the amplitude of an alpha oscillation (11 Hz). (c) Cortical column model. The values of the parameters are given in Tables 1 and 2. Multiple PAC combinations are present (see Figure 3). In all cases, the temporal dynamics of excitatory and inhibitory populations are depicted in red and blue, respectively.

Figure 2(a) shows a realization of the model where the phase of a 4 Hz oscillation modulates the amplitude of a 55 Hz oscillation. The parameter values used in this simulation were *τ*_*E*_ = 0.0032 s, *τ*_*I*_ = 0.0052 s, *p*_*E*_ = 0.6 + 0.1cos⁡(8*πt*), *p*_*I*_ = 0, Γ_*EE*_ = 2.4, Γ_*EI*_ = 2, Γ_*IE*_ = 2.1, Γ_*II*_ = 0, *r* = 4, *S*_0_ = 1, and *v*_0_ = 1. Additional simulations showed [21] that the amplitude, frequency, and phase-locking characteristics of the PAC activity generated were dependent on the strength of the connectivity parameters and on the amplitude and mean value of the low frequency input signal. It was possible to tune the parameters of the model to produce different frequencies of activity phase-locked to different phases of the theta rhythm [21].

### 5.2. The Jansen-Rit Model of a Cortical Column

The Jansen-Rit model of a cortical column [62] comprises three neuronal populations (Figure 1(b)): pyramidal cells, excitatory interneurons, and inhibitory interneurons. The model is mathematically described by a system of second-order differential equations:

(6) d2x0tdt2=−2adx0tdt−a2x0t+AaSx1t−x2t,d2x1tdt2=−2adx1tdt−a2x1t+Aap+Γ2SΓ1x0t,d2x2tdt2=−2bdx2tdt−b2x2t+BbΓ4SΓ3x0t,

where *x*_0_ is the excitatory postsynaptic potential (EPSP) that feeds into the two populations of interneurons and *x*_1_ and *x*_2_ are EPSP and inhibitory postsynaptic potentials (IPSP) that enter into the pyramidal cell population, respectively. Γ_1_, Γ_2_, Γ_3_, and Γ_4_ are the connection strengths between the populations. In this model, the electroencephalography (EEG) signal is considered to be proportional to *x*_1_(*t*) − *x*_2_(*t*).

Figure 2(b) shows a realization of model (6) where delta (3 Hz)-alpha (11 Hz) PAC is produced when considering an oscillatory input *p*. The parameter values used in this simulation were *A* = 3.25 mV, *B* = 22 mV, *p* = 200 + 50cos⁡(6*πt*), *a* = 100 s^−1^, *b* = 50 s^−1^, Γ_1_ = 135, Γ_2_ = 108, Γ_3_ = 33.75, Γ_4_ = 33.75, *r* = 0.56 mV^−1^, *S*_0_ = 5 s^−1^, and *v*_0_ = 6 mV.

Alternatively, EEG signals presenting PAC can be obtained by coupling multiple Jansen-Rit models (see Figures 5, 8, and 9 in [67]). In a more recent work [22], several Jansen-Rit models were also coupled and the cross-frequency transfer was studied in a setting where oscillators (generating the different rhythms) were coupled unidirectionally and thus the driving between them was passive. This study showed that this passive driving can also account for CFC in the brain as a result of the complex nonlinear dynamics of the underlying neuronal activity.

### 5.3. Cortical Column Model Comprising 4 Layers and 14 Neuronal Populations

A more complex neural mass model of the cortical column was recently proposed [79] in which 4 cortical layers and 14 neuronal populations are considered.  Figure 1(c) shows the model obtained by distributing four cell classes in four cortical layers (L2/3, L4, L5, and L6). This produced 14 different neuronal populations, since not all cell classes are present in every layer [80]. Excitatory neurons were either regular spiking (RS) or intrinsically bursting (IB) ones, and inhibitory neurons were either fast-spiking (FS) or low-threshold spiking (LTS) neurons.

The model is based on the Jansen-Rit model and the dynamics of the average PSP in each neuronal population *x*_*m*_ is obtained by solving a system of 14 second-order differential equations:

(7) d2xmtdt2=−2kmbmdxmtdt−km2xmt+Gmkmpm+∑n=114ΓnmSxnt,

where *n* = 1,…, 14, and *m* = 1,…, 14. The populations are numbered from 1 to 14 following the order: [L2RS, L2IB, L2LTS, L2FS, L4RS, L4LTS, L4FS, L5RS, L5IB, L5LTS, L5FS, L6RS, L6LTS, L6FS]. Layer 2/3 was labelled as 2. As can be seen in (7), neuronal populations interact via the connectivity matrix Γ_*nm*_ (Figure 1(d)). External inputs are accounted for via *p*_*m*_ which can be any arbitrary function including white noise [62]. The “damping” parameter *b*_*m*_ critically determines the behavior of the system. For *b*_*m*_ = 1 (which corresponds to the Jansen-Rit model) an individual population is not capable of oscillating, and it is the presence of interpopulation connections (nonzero Γ_*nm*_, *n* ≠ *m*) that produces oscillatory behavior that mimics observed EEG signals. To account for the possibility of an oscillatory population [78, 81] a nonzero value for *b*_*m*_ was used.

**Figure 3:**
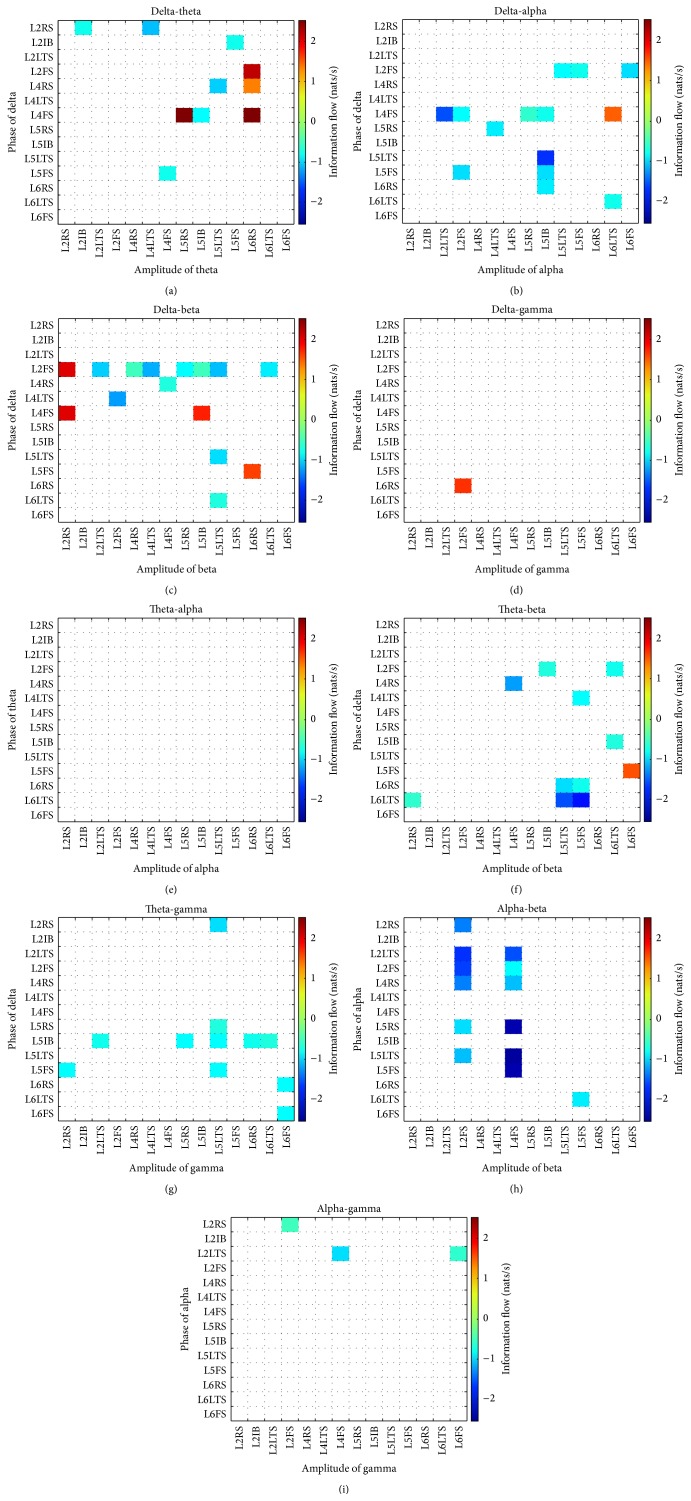
Phase-amplitude coupling (PAC) corresponding to the simulation presented in Figure 2(c). Nonsignificant values were set to zero and are depicted in white. (a) Delta-theta, (b) delta-alpha, (c) delta-beta, (d) delta-gamma, (e) theta-alpha, (f) theta-beta, (g) theta-gamma, (h) alpha-beta, and (i) alpha-gamma.

Figure 2(c) presents the temporal evolution of the average PSP in each neuronal population. Time series colored in red correspond to excitatory PSP (EPSP) whereas inhibitory PSP (IPSP) are presented in blue. As seen in the figure, both EPSP and IPSP present the characteristic “waxing and waning” (i.e., amplitude modulation) observed in real EEG signals. Parameters values are presented in Tables 1 and 2. To quantify the PAC phenomenon, a causality measure between time series, the information flow [82], was computed using phases and amplitudes of the signals shown in Figure 2(c).  Figure 3 shows the information flow from the phase to the amplitude for nine different combinations of phases and amplitudes: delta-theta, delta-alpha, delta-beta, delta-gamma, theta-alpha, theta-beta, theta-gamma, alpha-beta, and alpha-gamma. A negative value of the information flow means that the phase tends to stabilize the amplitude whereas a positive value means that the phase tends to make the amplitude more uncertain. An exploratory analysis of the influence of the parameters on PAC showed that changes in external input and time constants produced theta-gamma PAC values higher than alpha-gamma PAC, whereas changes in connectivity produced higher alpha-gamma PAC values. Additional information can be found in [79].

## 6. Conclusions

In conclusion, we have shown that PAC is a diverse phenomenon, not restricted to the theta-gamma coupling in the hippocampus. In order to model the complexity of the PAC phenomenon, which is hypothesized to bridge local and global scales in the brain [12, 15], reduced models of neuronal activity such as NMMs are needed, since detailed models are computationally expensive and their results are difficult to interpret due to the high number of variables and parameters involved. An open problem to be explored with NMMs is how the different PAC combinations are related.

While both types of models reviewed here, detailed models and NMMs, are capable of generating signals reflecting PAC, only in a few studies a quantitative measure of the phenomenon has been provided. This is probably related to the lack of a gold standard for PAC detection, which has resulted in the development of numerous methods.

The computational models summarized here focused on the mechanistic generation of the PAC phenomenon. NMMs are simple (in the sense of the few variables and parameters involved) but complex (in the sense of the richness of the dynamics they can generate) enough to approach important questions related to the functional role of the PAC phenomenon.

## Figures and Tables

**Table 1:** Values and physiological interpretation of the parameters.

Parameter (units)	Interpretation	Value
*G*(mV)	Gain	*G*_1_ = 3.25, *G*_2_ = 3.25, *G*_3_ = 30, *G*_4_ = 10, *G*_5_ = 3.25, *G*_6_ = 30, *G*_7_ = 10, *G*_8_ = 3.25, *G*_9_ = 3.25, *G*_10_ = 30, *G*_11_ = 10, *G*_12_ = 3.25, *G*_13_ = 30, and *G*_14_ = 10

*k* (s^−1^)	Reciprocal of time constant	*k*_1_ = 60, *k*_2_ = 70, *k*_3_ = 30, *k*_4_ = 350, *k*_5_ = 60, *k*_6_ = 30, *k*_7_ = 350, *k*_8_ = 60, *k*_9_ = 70, *k*_10_ = 30, *k*_11_ = 350, *k*_12_ = 60, *k*_13_ = 30, and *k*_14_ = 350

*p*	External input	*p*_*i*_ = 0 for *i* ≠ {5,7}, *p*_5_ = 500, and *p*_7_ = 150

*b*	Damping coefficient	*b* = 0.06 for all populations

*S*_0_ (s^−1^)	Maximum firing rate	*e*_0_ = 5 for all populations

*v*_0_ (mV)	Position of the sigmoid function	*v*_0_ = 6 for all populations

*r* (mV^−1^)	Steepness of the sigmoid function	*r* = 0.56 for all populations

**Table 2:** Standard values of the connectivity matrix Γ_*nm*_.

		L2/3				L4			L5				L6		
		RS	IB	LTS	FS	RS	LTS	FS	RS	IB	LTS	FS	RS	LTS	FS
L2/3	RS	25	10	10	15	0	25	30	0	0	0	0	0	0	0
	IB	10	25	5	5	0	0	0	0	0	0	0	0	0	0
	LTS	−10	−8	−15	−10	0	0	0	−20	−25	0	0	0	0	0
	FS	−15	−10	0	−15	0	0	0	−20	−25	0	0	0	0	0

L4	RS	12	10	0	0	15	30	25	8	18	0	0	0	0	0
	LTS	−20	0	0	0	−20	−25	−10	0	0	0	0	0	0	0
	FS	−42	0	0	0	−22	0	25	0	0	0	0	0	0	0

L5	RS	0	0	0	0	0	0	0	12	0	22	18	25	0	0
	IB	0	0	0	0	0	0	0	10	10	22	18	25	0	0
	LTS	0	0	0	0	0	0	0	−10	−10	−10	−20	−25	0	−30
	FS	0	0	0	0	0	0	0	−19	−19	−17	−15	0	0	0

L6	RS	0	0	0	0	45	0	10	0	0	0	0	15	10	10
	LTS	0	0	0	0	0	0	0	0	0	0	0	−11	−10	−8
	FS	0	0	0	0	0	0	0	0	0	0	0	−20	0	−15
